# Real-World Evaluation of ANA Testing Pathways: Serological Incompleteness and Comparison of Stepwise Versus Upfront Parallel Reflex Strategies

**DOI:** 10.3390/biomedicines14081815

**Published:** 2026-08-12

**Authors:** Raffaele Radice, Francesca Carreras, Chiara Corrado, Michela Salvatici, Delia Francesca Sansico, Barbara Bianchi, Monica Gaimarri, Lucia La Sala, Elena Dozio, Lorenzo Drago

**Affiliations:** 1 UOC Laboratory of Clinical Medicine with Specialized Areas, IRCCS MultiMedica Hospital, 20138 Milan, Italy; raffaele.radice@unimi.it (R.R.); francesca.carreras@multimedica.it (F.C.); chiara.corrado@multimedica.it (C.C.); michela.salvatici@multimedica.it (M.S.); deliafrancesca.sansico@multimedica.it (D.F.S.); barbara.bianchi@multimedica.it (B.B.); monica.gaimarri@multimedica.it (M.G.); 2 Department of Biomedical Sciences for Health, University of Milan, 20133 Milan, Italy; 3 Laboratory of Diabetology, Dysmetabolic Diseases, and Advanced Clinical Research—IRCCS MultiMedica, IRCCS MultiMedica, Via Fantoli 16/15, 20138 Milan, Italy; 4 Experimental Laboratory for Research on Organ Damage Biomarkers, Istituto Auxologico Italiano, IRCCS, 20133 Milan, Italy; 5 Clinical Microbiology and Microbiome Laboratory, Department of Biomedical Sciences for Health, University of Milan, 20133 Milan, Italy

**Keywords:** antinuclear antibodies, ANA-IIF, reflex testing, autoimmune diagnostics, ENA screening

## Abstract

**Background/Objectives**: Antinuclear antibody testing by indirect immunofluorescence on HEp-2 cells (ANA-IIF) remains the reference test for systemic autoimmune rheumatic diseases (SARD). However, complete serological characterization requires integration with extractable nuclear antigen (ENA) screening, antigen-specific solid-phase assays (SPA), and confirmatory indirect immunofluorescence (IIF) tests. This study aimed to: (1) quantify real-world serological pathway incompleteness in ANA testing performed outside a structured reflex strategy; (2) compare simulated stepwise and upfront parallel reflex approaches in terms of serological yield, laboratory workload, and costs. **Methods:** We retrospectively analyzed 2207 consecutive patients referred for ANA-IIF testing between July 2025 and April 2026. Serological incompleteness was defined as non-completion of the predefined Regione Lombardia reflex framework. A fully characterized subgroup of 514 patients, defined by complete reflex-serological assessment, was used to compare a stepwise strategy, in which ANA-IIF positivity triggered downstream testing, with an upfront parallel strategy, in which ANA-IIF, ENA screening, and anti-double-stranded DNA (anti-dsDNA) testing were performed upfront. **Results:** ANA-IIF was positive in 913/2207 patients (41.4%). Overall, 508 patients (23.0%) had at least one missing follow-up assay expected under the reflex framework. In the fully characterized subgroup, the upfront parallel strategy increased the number of patients with at least one fully characterized autoantibody finding from 15 to 22 (+1.4%), but required 657 additional tests and an estimated incremental cost of approximately €2592. **Conclusions**: ANA-IIF testing outside a structured reflex pathway frequently resulted in incomplete serological assessment. Although the upfront parallel strategy increased serological yield, it required substantially greater laboratory workload and costs than the stepwise approach. Further studies are needed to define the optimal balance between serological yield, clinical relevance, and cost-effectiveness.

## 1. Introduction

The laboratory evaluation of systemic autoimmune rheumatic diseases (SARDs) relies on the detection and characterization of autoantibodies, which are central to clinical decision-making. Antinuclear antibodies detected by indirect immunofluorescence on HEp-2 cells (ANA-IIF) remain the reference screening assay because of their high sensitivity and ability to provide clinically informative fluorescence patterns [1]. However, ANA-IIF alone does not ensure complete serological characterization. Its limited specificity, the occurrence of ANA positivity in healthy individuals and non-rheumatic conditions, and its reduced ability to detect selected clinically relevant autoantibodies support the integration of Extractable Nuclear Antigen (ENA) screening, antigen-specific solid-phase assays (SPAs), and confirmatory immunofluorescence (IIF) assays [2,3].

In routine practice, these tests are often ordered independently rather than within structured diagnostic pathways. This may lead to heterogeneous testing patterns and incomplete or delayed follow-up investigations, even when additional testing is analytically indicated. As a result, relevant serological information may be missed, leading to incomplete characterization of the autoantibody profile.

To reduce this variability, autoimmunity laboratories are increasingly adopting reflex testing strategies, in which follow-up investigations are performed according to predefined algorithms integrated into the laboratory workflow [4]. These strategies connect screening assays, antigen-specific characterization, and confirmatory testing, ideally on the same sample, thereby improving pathway completion and reducing turnaround times.

**Figure 1:**
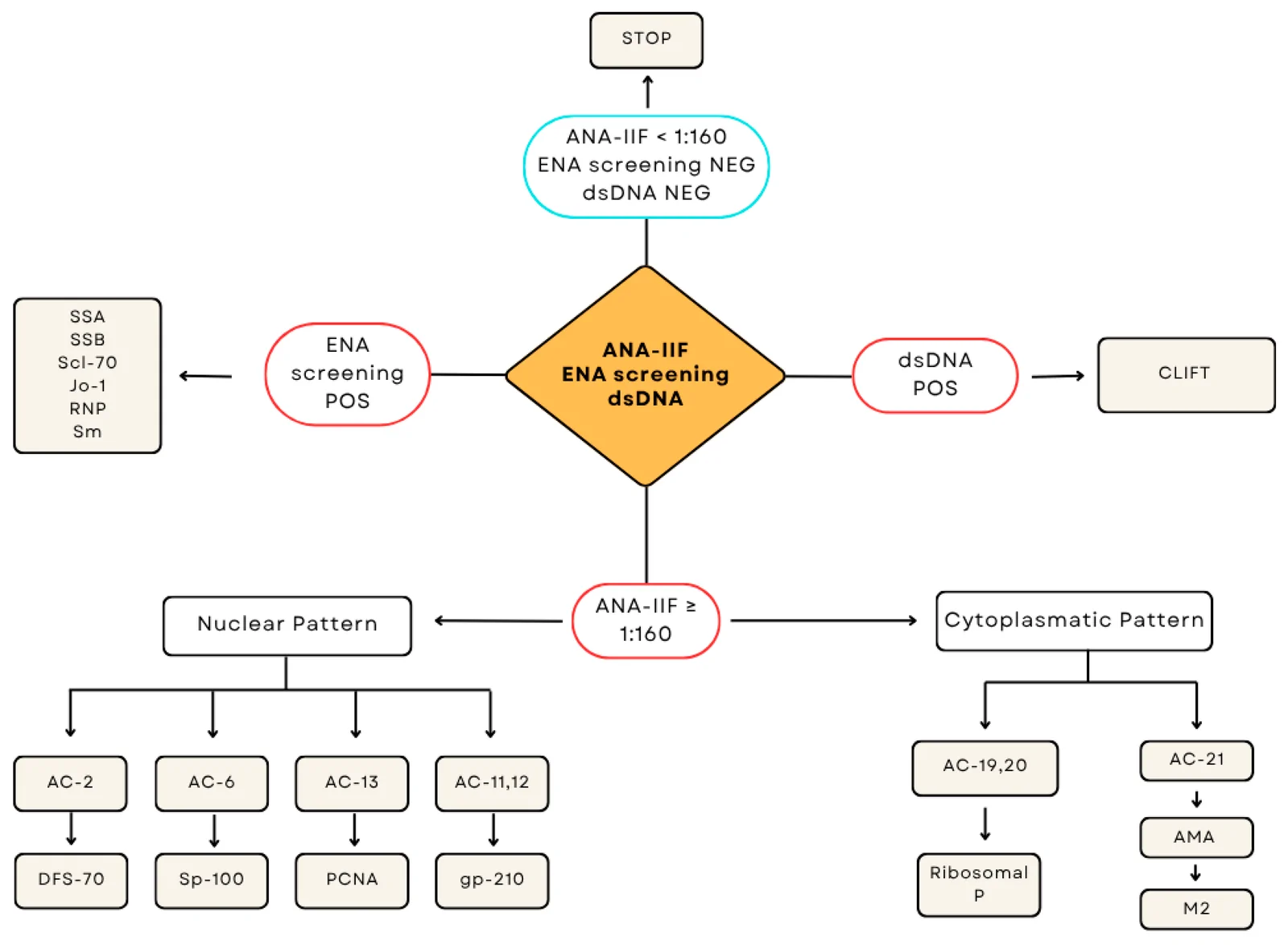
Schematic overview of the Regione Lombardia upfront parallel model, in which ANA-IIF, ENA screening, and anti-dsDNA testing are performed as initial entry tests. Further confirmatory and second-level assays are then guided by SPA-positive results and by interpretation of the ANA-IIF fluorescence pattern. Abbreviations: ANA-IIF, antinuclear antibody indirect immunofluorescence; ENA, extractable nuclear antigen; dsDNA, double-stranded DNA; CLIFT, Crithidia luciliae immunofluorescence test; SSA, Sjögren’s-syndrome-related antigen A; SSB, Sjögren’s-syndrome-related antigen B; RNP, ribonucleoprotein; Sm, Smith antigen; DFS70, dense fine speckled 70 kDa; PCNA, proliferating cell nuclear antigen; AMA, antimitochondrial antibody; AC, anti-cell pattern code; POS, positive; NEG, negative.

Within the regional laboratory network of Lombardia, Italy, a reflex strategy has been implemented. This strategy includes the simultaneous performance of ANA-IIF, ENA screening, and anti-dsDNA testing at baseline, followed by antigen-specific SPA and/or confirmatory IIF when indicated (Figure 1) [5]. The overall model is based on established associations between ANA-IIF patterns, ENA screening results, anti-dsDNA results, and targeted follow-up testing [4,6]. Performing the three baseline tests upfront may maximize analytical detection, although it also increases the number of tests performed [7,8].

**Figure 2:**
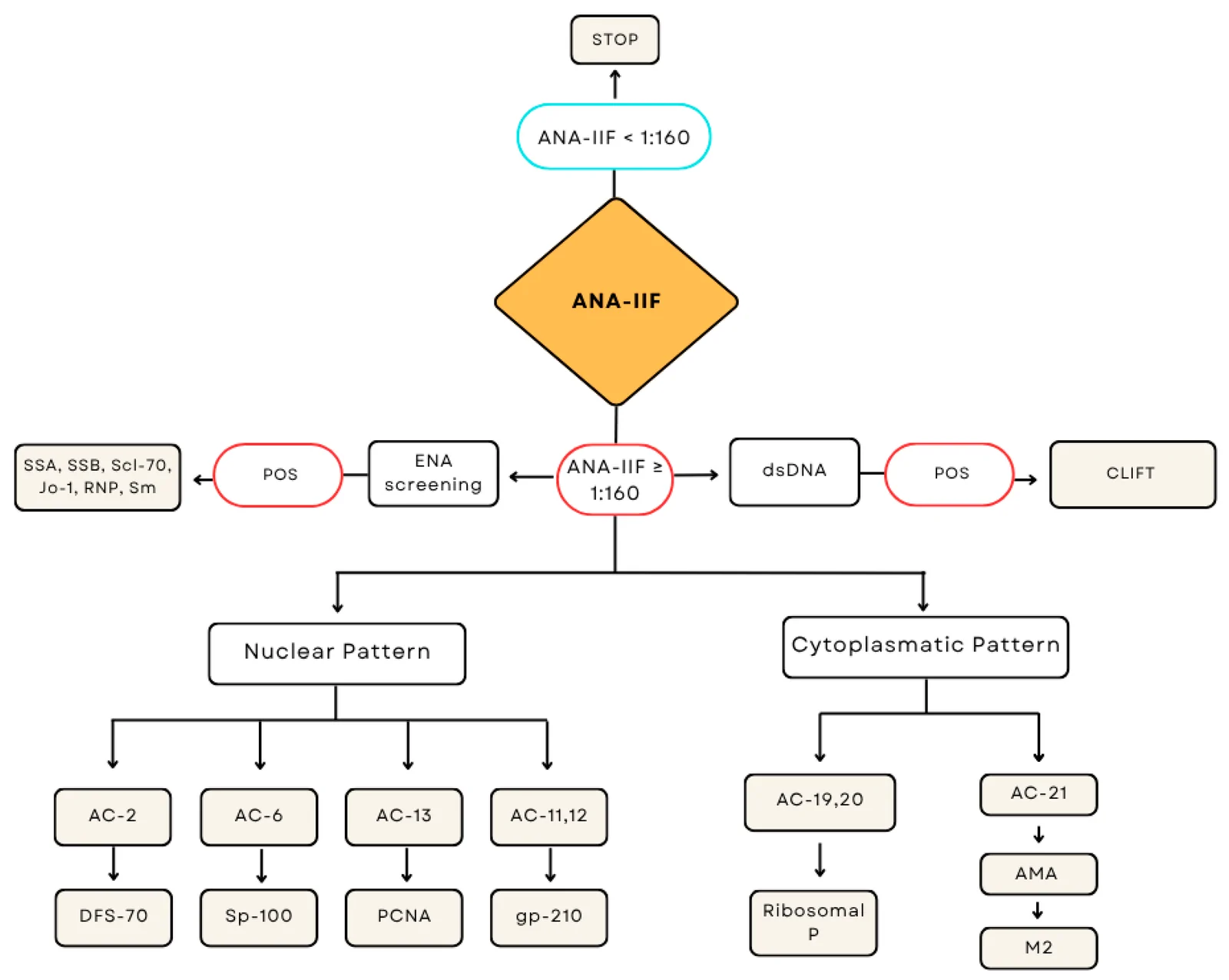
Schematic overview of the stepwise model, in which ENA screening and anti-dsDNA testing are performed only after a positive ANA result, defined as a titer ≥ 1:160. Abbreviations: ANA-IIF, antinuclear antibody indirect immunofluorescence; ENA, extractable nuclear antigen; dsDNA, double-stranded DNA; CLIFT, Crithidia luciliae immunofluorescence test; SSA, Sjögren’s-syndrome-related antigen A; SSB, Sjögren’s-syndrome-related antigen B; Scl-70, topoisomerase I; Jo-1, histidyl-tRNA synthetase; RNP, ribonucleoprotein; Sm, Smith antigen; AC, anti-cell pattern code; DFS-70, dense fine speckled 70 kDa; PCNA, proliferating cell nuclear antigen; AMA, antimitochondrial antibody; POS, positive.

This trade-off is particularly relevant given the issue of test appropriateness, which remains a major concern in autoimmune diagnostics [9,10]. A substantial proportion of ANA requests are performed in patients with low pre-test probability, especially in primary care settings. In this context, broader strategies may increase analytical detection but also testing burden and clinically non-specific findings [11]. Conversely, a stepwise strategy, in which only ANA-IIF triggers downstream investigations, might improve resource utilization (Figure 2).

Previous regional initiatives have attempted to standardize autoimmune serology through structured diagnostic algorithms. Bonaguri et al. described the implementation of a regional diagnostic algorithm for autoimmune rheumatic diseases, demonstrating improved appropriateness and harmonization of autoantibody testing across participating laboratories [12].

Despite the increasing adoption of reflex testing strategies, little is known about the frequency of incomplete serological follow-up after ANA-IIF testing performed outside a structured reflex pathway.

Using the Regione Lombardia ANA reflex framework as the reference model, we aimed first to quantify the frequency of incomplete serological pathways after ANA-IIF testing performed outside a structured reflex strategy, and second, to compare stepwise and upfront parallel reflex approaches in terms of serological yield, laboratory workload, and costs. Importantly, serological incompleteness reflects laboratory pathway completion within the Regione Lombardia reflex framework and should not be interpreted as a measure of diagnostic accuracy or clinical outcomes.

## 2. Materials and Methods

### 2.1. Study Population

A retrospective observational study was conducted using laboratory data from 2207 consecutive, unselected patients referred to the Laboratory Medicine Unit of IRCCS MultiMedica, Milan, Italy, for ANA-IIF testing between July 2025 and April 2026. Of these, 1294 samples (58.6%) were ANA-IIF negative and 913 (41.4%) were ANA-IIF positive. Patients were referred for a broad range of clinical indications potentially prompting autoimmune serological testing. Clinical indications are summarized in Table 1.

Patients were referred by general practitioners, rheumatologists, or other medical specialists for suspected autoimmune conditions. The only inclusion criterion was the presence of an ANA-IIF request. No exclusion criteria were applied. The study was conducted in accordance with the Declaration of Helsinki and was approved by the Comitato Etico Territoriale Lombardia 5, Rozzano, Italy (protocol no. 378/24; approved on 23 July 2024). Samples were anonymized before analysis, and written informed consent was obtained in accordance with institutional and ethical requirements.

**Figure 3:**
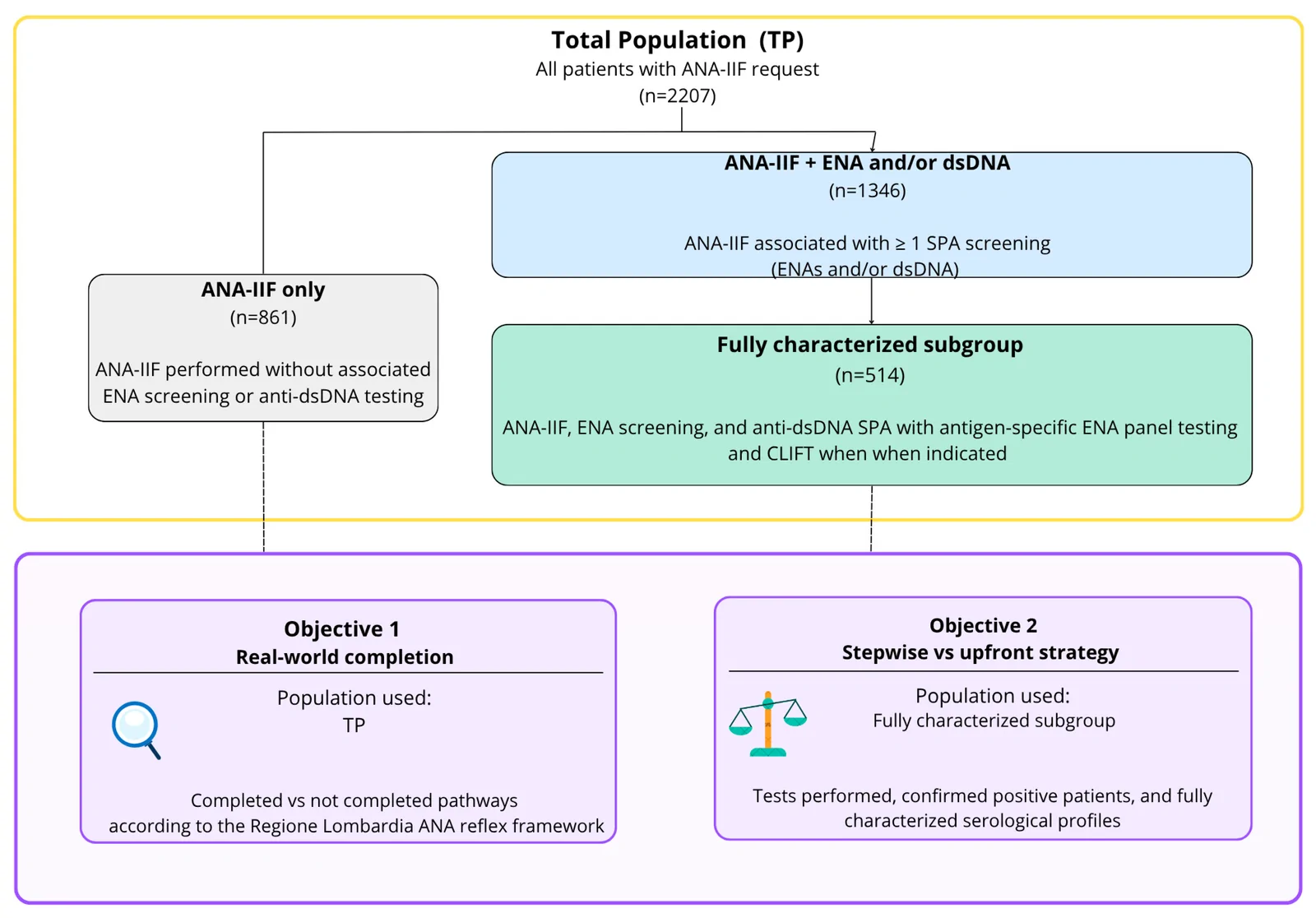
Flowchart showing the total ANA-IIF request population, the derived testing subgroups, and the two study objectives: real-world pathway completion and comparison of stepwise versus upfront parallel reflex strategies. Abbreviations: TP, total population; ANA-IIF, antinuclear antibody indirect immunofluorescence; ENA, extractable nuclear antigen; dsDNA, double-stranded DNA; SPA, solid-phase assay; CLIFT, Crithidia luciliae immunofluorescence test.

Overall, 861 patients (39.0%) underwent ANA-IIF only, whereas 1346 (61.0%) underwent ANA-IIF associated with ENA screening and/or anti-dsDNA testing. Simultaneous baseline availability of ANA-IIF, ENA screening, and anti-dsDNA testing was present in 529 patients (24.0%); after the exclusion of 15 patients with positive or borderline ENA screening but unavailable antigen-specific ENA characterization, the fully characterized subgroup included 514 patients (23.3%). An overview of these distributions is provided in Figure 3.

### 2.2. Laboratory Assays

ANA-IIF was performed using the NOVA Lite HEp-2 ANA kit (Inova Diagnostics, San Diego, CA, USA). Serum samples were screened at 1:160, adopted as the positivity cut-off. IIF assays were processed using QUANTA-Lyser^®^ 3000 (Inova Diagnostics, San Diego, CA, USA). Results were reported as positive or negative and included endpoint titers and fluorescence patterns, classified according to ICAP nomenclature [6]. Patterns and titers were evaluated using a manual LED fluorescence microscope (H550L, Nikon, Tokyo, Japan) by two expert clinical pathologists; discrepancies were resolved by consensus.

ENA screening was performed by FEIA on the EliA™ system (Thermo Fisher Scientific, Uppsala, Sweden), run on a Phadia 250 instrument (Thermo Fisher Scientific, Uppsala, Sweden), according to the manufacturer’s instructions. Results were expressed as a ratio: values ≥ 1.0 were considered positive, values between 0.7 and 1.0 borderline, and values < 0.7 negative. The ENA screening panel included SSA, SSB/La, U1-RNP, Sm, centromere B, Scl-70, and Jo-1. Single antigen-specific SPAs were performed on the same platform and included all ENA specificities present in the screening assay, with separate determination of the SSA/Ro60 and SSA/Ro52 subcomponents, as well as anti-dsDNA, anti-ribosomal *P*, anti-PCNA, and anti-DFS70. Borderline or positive anti-dsDNA SPA results were further assessed by CLIFT (NOVA Lite^®^, Inova Diagnostics, San Diego, CA, USA), which was considered the predefined confirmatory IIF endpoint for anti-dsDNA reactivity within this laboratory algorithm [13].

AMA-IIF was performed on rodent tissue sections (NOVA Lite^®^, Inova Diagnostics, San Diego, CA, USA). Additional liver-specific antigen-specific SPA were assessed using EUROLINE Liver Profile (EUROIMMUN, Lübeck, Germany), including gp210, AMA-M2, M2-3E (BPO), and Sp100. AMA-M2 and M2-3E were considered antigen-specific follow-up assays for antimitochondrial reactivity [14,15,16].

### 2.3. Study Design, Analytical Population, and Endpoints

Clinical and laboratory data were collected in a structured database, including ANA-IIF titers and patterns, ENA screening results, single antigen-specific ENA results, anti-dsDNA results, CLIFT results, test dates, and clinical indication when available.

The study was structured around two analytical populations. The total population (TP; *n* = 2207) was used to assess real-world serological incompleteness, defined as non-completion of the predefined ANA reflex framework adopted by the Regione Lombardia laboratory network. The framework links ANA-IIF results, ENA screening, anti-dsDNA testing, and selected ICAP patterns to expected follow-up assays. Therefore, incompleteness was defined as non-completion of this laboratory pathway, not as an absolute clinical standard. Triggers included ANA-IIF positivity requiring ENA screening; positive or borderline ENA screening requiring antigen-specific ENA characterization; positive or borderline anti-dsDNA SPA requiring CLIFT; and selected ANA-IIF patterns requiring targeted tests: AC-21 for AMA-IIF, and when indicated, antigen-specific antimitochondrial assays; AC-6 for anti-Sp100; AC-11/AC-12 for anti-gp210; and AC-19/AC-20 for anti-ribosomal *P* [17,18].

Furthermore, the fully characterized subgroup of 514 patients was used to simulate and compare the stepwise and upfront parallel reflex strategies. This subgroup included patients with complete availability of baseline ANA-IIF, ENA screening, and anti-dsDNA testing, together with all reflex-confirmatory assays required in the presence of positive or borderline screening results. Patients lacking any required second-level characterization were excluded from the simulation analysis.

To assess the potential for selection bias and evaluate the representativeness of the fully characterized subgroup, its baseline laboratory and referral characteristics were compared with those of the remaining study cohort. The parameters used for comparison included ANA-IIF result (positive or negative), ANA-IIF titer, ANA-IIF pattern, and clinical indication for testing. These comparisons are reported in Supplementary Table S1.

Stepwise and upfront parallel strategies were simulated in the fully characterized subgroup. In the stepwise strategy, ANA-IIF positivity, defined as a titer ≥ 1:160, triggered ENA screening, anti-dsDNA SPA, and downstream characterization when indicated (Figure 2). In the upfront parallel strategy, ANA-IIF, ENA screening, and anti-dsDNA SPA were assumed to be performed simultaneously in all patients, followed by antigen-specific SPA and/or confirmatory IIF when indicated (Figure 1). A patient was considered fully characterized when any positive or borderline screening-level result underwent the corresponding antigen-specific or confirmatory testing required by the reflex framework. For each strategy, we estimated tests performed, screening-level signals, fully characterized serological findings, unique characterized patients, and additional serological yield. Accordingly, the present study was designed to evaluate laboratory pathway completion rather than diagnostic accuracy. Because clinical diagnoses and patient outcomes were not available, diagnostic performance measures such as sensitivity, specificity, positive predictive value, and negative predictive value could not be assessed.

The primary endpoint was the proportion of patients completing the predefined reflex pathway after a laboratory trigger requiring further testing. Secondary endpoints, assessed in the fully characterized subgroup, included the serological yield, laboratory workload, and estimated economic impact of stepwise versus upfront parallel strategies.

### 2.4. Cost Analysis

An exploratory cost analysis was performed in the fully characterized subgroup to compare the economic burden of the stepwise and upfront parallel strategies. The analysis included the core tests differentiating the two approaches (ANA-IIF, ENA screening, anti-dsDNA SPA, antigen-specific ENA assays, and CLIFT confirmation when indicated), whereas pattern-driven follow-up tests were excluded. Unit costs were adapted from the reimbursement-based model reported by Bizzaro et al. [8]: €4 for ANA-IIF, €4 for ENA screening, €4 for the anti-dsDNA pathway, and €32 for the eight-antigen ENA panel. Total costs were calculated by multiplying the number of tests generated by each strategy by the corresponding unit value, and the incremental cost was defined as the difference between the upfront parallel and stepwise approaches. This analysis should be interpreted as an exploratory reimbursement-based estimate rather than as a direct measure of laboratory production costs.

### 2.5. Statistical Analysis

Categorical variables (clinical indications, screening results, and pathway incompleteness rates) were summarized and reported as absolute frequencies (*n*) and percentages (%). Cross-tabulations were used to determine the rate of screening-level concordance and discordance between ANA-IIF and the solid-phase assay results within the fully characterized subgroup. To evaluate the stepwise and upfront parallel reflex strategies, a deterministic simulation model was applied to the fully characterized cohort (*n* = 514). The two approaches were compared descriptively in terms of screening-level detection, antigen-specific characterization, and laboratory workload (expressed as total tests and tests per patient). Because the two simulated strategies were applied to the same patient cohort, paired comparisons of categorical outcomes were performed using the exact McNemar’s test, and differences in proportions were reported with 95% confidence intervals (95% CI). A two-sided *p*-value < 0.05 was considered statistically significant. All analyses and data management were conducted using Microsoft Excel 2016 (Microsoft Corporation, Redmond, WA, USA).

## 3. Results

### 3.1. Serological Incompleteness in Total Population

Among the 913 ANA-IIF-positive patients, 400 (43.8%) did not undergo ENA screening at baseline. Among the 400 ANA-IIF-positive patients without ENA screening at baseline, only 26 subsequently underwent ENA screening during the available laboratory follow-up period. Of these, 3/26 had a positive ENA screening result.

Among the ANA-IIF-positive patients, 62 exhibited a cytoplasmic reticular, AMA-like pattern (AC-21). However, 30 of these patients (48.4%) did not undergo AMA-IIF testing, and only 9 underwent confirmatory AMA-M2 testing. In addition, 59 patients displayed cytoplasmic patterns compatible with anti-ribosomal *P* reactivity (AC-19/AC-20). Of these, only 9 underwent antigen-specific SPA testing, whereas 50 patients (84.7%) did not receive the corresponding follow-up assay. In addition, 4 patients displayed the AC-6 pattern, suggestive of anti-Sp100 reactivity, of whom 3 (75.0%) did not undergo anti-Sp100 testing. Similarly, all 3 patients with AC-11/AC-12 patterns, suggestive of anti-gp210 reactivity, remained without anti-gp210 testing.

Among the 92 samples with positive or borderline ENA screening, antigen-specific ENA characterization was performed in 37 cases when considering both baseline and subsequent testing. Consequently, 55 patients (59.8%) remained without antigen-specific characterization throughout the available observation period.

Overall, 508 patients (23.0% of the total population) did not undergo at least one follow-up test expected under the predefined reflex framework, resulting in incomplete serological pathways.

The different categories of serological incompleteness are detailed in Table 2.

### 3.2. Stepwise Versus Upfront Parallel Strategy in Fully Characterized Subgroup

As shown in Supplementary Table S1, the fully characterized subgroup showed broadly similar distributions of ANA-IIF status, ANA-IIF titer, ANA-IIF pattern, and clinical indication for testing compared with the remaining study cohort. This descriptive comparison suggests comparability between the two groups for the variables assessed. In the fully characterized subgroup, 222 patients were ANA-IIF-positive and 292 were ANA-IIF-negative. Before simulating the two strategies, screening-level discordance was assessed within this subgroup to estimate the amount of antigen-based reactivity that would not be captured by an ANA-IIF-gated approach. Overall, 47/514 patients (9.1%) showed at least one positive or borderline screening-level result by ENA screening and/or anti-dsDNA SPA. These included 31/222 ANA-IIF-positive patients (14.0%) and 16/292 ANA-IIF-negative patients (5.5%). Thus, ANA-IIF-negative screening-level reactivity accounted for 3.1% of the fully characterized subgroup and represented the fraction of patients potentially missed by a strictly ANA-IIF-restricted stepwise strategy.

The stepwise strategy identified 31 patients (6.0%) with at least one screening-level serological signal: 19 (3.7%) with positive or borderline ENA screening and 13 (2.5%) with positive or borderline anti-dsDNA. After characterization, 13 patients (2.5%) had ENA-specific autoantibodies and 3 (0.6%) CLIFT-positive anti-dsDNA findings, corresponding to 15 unique patients (2.9%) with at least one fully characterized serological finding. The upfront parallel strategy identified 47 patients (9.1%) with at least one screening-level signal: 27 (5.3%) with positive or borderline ENA screening and 22 (4.3%) with positive or borderline anti-dsDNA. After characterization, 18 patients (3.5%) had ENA-specific autoantibodies and 5 (1.0%) had CLIFT-positive anti-dsDNA findings, resulting in 22 patients (4.3%) with at least one fully characterized serological finding.

Compared with the stepwise approach, the upfront parallel strategy yielded 16 additional patients with screening-level signals (3.1% of the subgroup; 47/514 vs. 31/514; 95% CI: 1.62% to 4.61%; *p* < 0.001, McNemar’s test) and 7 additional patients with fully characterized autoantibody findings (incremental gain, 1.4%; 22/514 vs. 15/514 95% CI: 0.47% to 2.25%; *p* = 0.016, McNemar’s test). All 7 additional patients were ANA-IIF negative and would therefore not have undergone downstream testing under a strictly ANA-IIF-gated stepwise strategy (Table 3).

Among the ENA screening-positive or borderline samples, the most frequent ENA specificities were Ro60 (*N* = 8), Ro52 (*N* = 6), and U1-RNP (*N* = 5), followed by Scl-70 (*N* = 2), La (*N* = 1), centromere B (*N* = 1), and borderline Jo-1 reactivity (*N* = 1). Among the ANA-IIF-negative patients, ENA specificities missed by an ANA-IIF-restricted stepwise strategy were mainly Ro60 (*N* = 3), followed by Ro52 (*N* = 1), Scl-70 (*N* = 1), and borderline Jo-1 (*N* = 1). Clinical follow-up data were not available for this retrospective laboratory-based study. Therefore, it was not possible to determine whether these additional serological findings resulted in changes in clinical diagnosis or patient management.

### 3.3. Exploratory Economic Analysis

From a workload perspective, the stepwise and upfront parallel approaches generated 1123 and 1780 tests, respectively, corresponding to 2.18 and 3.46 tests per patient. The upfront parallel approach therefore required 657 additional tests.

Using the reimbursement-based unit costs defined in the Methods, section the estimated total cost was €4440 for the stepwise approach and €7032 for the upfront parallel approach, corresponding to an incremental cost of €2592 (+58.4%; +€5.04 per patient) (Table 4). This increase was mainly attributable to the systematic use of ENA screening and anti-dsDNA testing in all patients, including ANA-IIF-negative samples. As pattern-driven follow-up assays were excluded, these findings should be interpreted as the incremental economic burden of upfront ENA/dsDNA testing rather than as a comprehensive cost-effectiveness estimate.

## 4. Discussion

The present study highlighted a substantial degree of serological pathway incompleteness in routine ANA-IIF testing performed outside a structured reflex strategy. According to the predefined Regione Lombardia reflex framework, 508 out of 2207 patients (23.0%) did not undergo at least one follow-up investigation expected after a laboratory trigger. Although this definition of incompleteness was based on a predefined laboratory algorithm rather than on clinical outcomes, the findings indicate that a considerable proportion of potentially informative serological data may remain unavailable in routine practice.

The main source of incompleteness was the absence of ENA screening after ANA-IIF positivity. Among the ANA-IIF-positive patients, 43.8% had not undergone ENA screening at baseline, and this proportion remained largely unchanged after the available follow-up period (41.0%). This finding is particularly relevant because ANA-IIF provides information on antibody titer and fluorescence pattern but does not define antigen specificity [19]. Consequently, failure to perform ENA screening may prevent complete characterization of the autoantibody response. Nevertheless, additional testing may reasonably be withheld in routine practice because of low clinical suspicion, previous external results, or the absence of features suggestive of systemic autoimmune rheumatic disease [20].

Serological incompleteness was not limited to ENA screening. Pattern-driven follow-up investigations were also frequently absent, particularly for AC-19/AC-20, AC-6, and AC-11/AC-12 patterns. This observation suggests that outside a structured reflex pathway, the interpretative information provided by ANA-IIF patterns does not consistently translate into targeted downstream investigations.

A further source of incompleteness involved positive or borderline ENA screening results that were not followed by antigen-specific characterization. In this subgroup, 59.8% of patients remained without characterization of individual ENA specificities during the available observation period.

Taken together, these findings suggest that serological incompleteness can occur at multiple stages of the diagnostic pathway, including failure to initiate second-level testing, failure to act upon ANA-IIF pattern information, and failure to complete antigen-specific characterization after a positive screening result.

These observations provide the rationale for structured reflex strategies, which aim to standardize downstream testing and reduce variability in pathway completion. However, the optimal design of such strategies remains uncertain, particularly regarding the balance between maximizing serological detection and minimizing unnecessary testing. To explore this issue, we compared an ANA-IIF-gated stepwise strategy with an upfront parallel approach.

The simulation of the two structured strategies showed a clear trade-off between serological completeness and resource utilization. The upfront parallel strategy increased the proportion of patients with at least one screening-level signal from 6.0% to 9.1% and the proportion of patients with at least one fully characterized serological finding from 2.9% to 4.3%. This corresponded to seven additional ANA-IIF-negative patients, resulting in an absolute incremental yield of 1.4%. These additional findings included five ENA-specific reactivities, predominantly anti-Ro/SSA antibodies, particularly anti-Ro60, and two CLIFT-positive anti-dsDNA results. Anti-Ro/SSA antibodies are clinically relevant biomarkers and represent one of the highest-weighted serological items in the classification criteria for primary Sjögren’s syndrome [21]. Consistently, a recent meta-analysis identified anti-Ro/SSA as the autoantibody specificity most frequently missed by ANA-gated testing strategies [22]. However, because clinical follow-up data were unavailable, it was not possible to determine whether these additional serological findings translated into new diagnoses or changes in patient management. Their clinical significance therefore remains uncertain. Previous studies have reported a low diagnostic yield of isolated ENA positivity in ANA-IIF-negative patients for newly diagnosed SARD [23,24,25].

The performance of an ANA-IIF-gated strategy could potentially be improved by using HEp-2000 substrates, which overexpress Ro60/SSA and may enhance the detection of anti-Ro60 reactivity, thereby reducing the proportion of Ro-positive cases missed by conventional HEp-2 ANA-IIF [26,27]. Beyond reducing laboratory workload, an ANA-IIF-gated stepwise strategy may also optimize the allocation of diagnostic resources. Tests avoided in patients with a low probability of clinically relevant autoimmunity could instead be redirected toward more informative investigations, including ICAP pattern-oriented antigen panels, disease-specific immunoblot assays, or additional confirmatory testing [28].

From an organizational and economic perspective, the upfront parallel strategy increased both laboratory workload and estimated expenditure by approximately 58% compared with the stepwise approach. Given the modest incremental serological yield observed, these findings emphasize the need to balance analytical completeness against resource utilization when designing reflex testing algorithms. In contrast, an ANA-IIF-gated stepwise strategy may preserve resources by avoiding systematic ENA screening and anti-dsDNA testing in ANA-IIF-negative samples. Previous studies have shown that ordering ENA testing in patients with a negative ANA result may increase healthcare expenditure without improving the detection of systemic autoimmune rheumatic diseases [23]. One possible use of the resources saved through this approach would be to strengthen targeted second-level testing in patients with informative ANA-IIF patterns. Pattern-oriented panels have been proposed, particularly for nucleolar and cytoplasmic patterns that are incompletely represented in conventional ENA panels [29]. However, this potential reallocation of resources was not directly evaluated in the present study.

Our findings are consistent with those of Gallo et al., who compared traditional, progressive, and combined ANA-screening algorithms in an unselected outpatient population [30]. Their combined approach achieved the highest antigen-specific autoantibody yield, whereas the progressive strategy showed greater economic efficiency. Similarly, our upfront parallel strategy increased serological yield but required substantially more tests and higher expenditure than the ANA-IIF-gated stepwise approach. Direct comparison remains limited by differences in assay composition, reflex rules, reimbursement systems, and effectiveness endpoints.

Although the present study was conducted in a single center and used the Regione Lombardia reflex framework as a reference model, its findings may provide a practical example for laboratories considering the implementation or revision of an ANA reflex strategy. Even within the same healthcare system, laboratories may serve populations with different referral patterns, disease prevalence, and pre-test probabilities, which may substantially affect the balance between serological yield, workload, and costs [31]. The baseline characteristics reported in Supplementary Table S1 may help external laboratories assess the comparability of their own patient population with that of the present study. Accordingly, our findings should be regarded as a framework for local evaluation and validation rather than as support for a universally applicable reflex algorithm. The optimal reflex model therefore remains uncertain, particularly because the clinical significance of the additional serological findings identified by broader testing strategies has yet to be fully established.

## 5. Conclusions

The present study identified substantial serological pathway incompleteness in routine ANA-IIF testing performed outside a structured reflex strategy, with nearly one-quarter of patients not completing at least one follow-up investigation expected under the predefined Regione Lombardia reflex framework. Structured reflex pathways may improve the completeness and standardization of autoimmune serology; however, their efficiency is likely to depend on the clinical setting and the pre-test probability of SARD. In our cohort, the upfront parallel strategy increased serological detection compared with an ANA-IIF-gated stepwise approach, but this gain was accompanied by substantially greater modeled laboratory workload and estimated expenditure. Conversely, the stepwise strategy showed greater laboratory efficiency, with the limited serological loss mainly involving anti-SSA/Ro reactivities. Because the clinical relevance of the additional findings was not assessed, it remains uncertain whether the greater resource investment associated with broader testing translates into meaningful clinical benefit. Further studies integrating laboratory findings with clinical diagnoses, patient management, and outcomes are needed to identify the most appropriate and sustainable reflex strategy across different clinical settings.

## 6. Limitations and Future Perspectives

This study has some limitations. First, the analysis was based exclusively on serological data; therefore, incomplete pathway completion and additional autoantibody findings cannot be directly equated with missed diagnoses, clinical benefit, or patient outcomes. The clinical significance of both incomplete serological characterization and the additional findings identified by the upfront parallel strategy remains uncertain and requires evaluation in clinically characterized populations. Observed pathway incompleteness may also have been influenced by previous testing performed outside the institution, disease severity and differences in pre-test probability. These factors were not systematically available in the laboratory dataset and could not be adjusted for in the present analysis.

The comparison between reflex strategies was performed in a fully characterized subgroup that may have been influenced by clinical suspicion and physician prescribing patterns, introducing potential selection bias. Consequently, the estimated serological yield of the simulated strategies may not fully reflect that of an unselected testing population.

In addition, serological incompleteness was defined relative to the predefined Regione Lombardia reflex framework adopted as the reference laboratory pathway in this study. This definition should not be interpreted as an absolute diagnostic standard, and alternative testing algorithms may be equally appropriate in different healthcare settings. Moreover, because the study population included many patients without a primary suspicion of systemic autoimmune rheumatic disease, incomplete pathway completion should not necessarily be interpreted as evidence of inappropriate clinical management.

Finally, the economic evaluation was limited to reimbursement-based laboratory costs and did not account for personnel, infrastructure, turnaround time, or downstream healthcare costs. Therefore, it represents a simplified estimate of laboratory resource utilization rather than a comprehensive health economic evaluation.

Prospective multicenter studies involving clinically characterized populations, different laboratory settings, analytical platforms, and alternative reflex algorithms are needed to confirm these findings. Future investigations should determine whether improved pathway completion and the additional serological findings identified by broader reflex strategies translate into meaningful diagnostic, prognostic, or therapeutic benefit, while also assessing their organizational impact and economic sustainability. Such evidence may also support the development of reimbursement models better aligned with the analytical complexity and resource requirements of modern autoimmune diagnostics.

## Figures and Tables

**Table 1:** ANA-IIF, antinuclear antibody testing by indirect immunofluorescence, on HEp-2 cells. Percentages are calculated using the total study population (*N* = 2207) as the denominator. Clinical indications were extracted from test request forms and categorized according to the predefined study classification.

Domain	Proportion (%)
ANA-IIF positive	41.4
ANA-IIF negative	58.6
**Clinical indication for ANA-IIF Testing**	
Musculoskeletal symptoms	27.1
Non-classifiable indications	14.4
Dermatological manifestations	8.9
Hepatological/gastroenterological conditions	8.1
Suspected systemic autoimmune disease	8.1
Pulmonary/respiratory conditions	8.1
Other indications	25.3

**Table 2:** Persistent serological incompleteness according to the predefined reflex framework during the available follow-up period.

Domain	Missing Follow-up Test	*n*/*N* (%)
ANA-IIF positive patients	ENA screening	374/913 (41.0%)
ANA-IIF AC-21 pattern	AMA-IIF	30/62 (48.4%)
ANA-IIF AC-6 pattern	Anti-Sp100	3/4 (75.0%)
ANA-IIF AC-11/AC-12 patterns	Anti-gp210	3/3 (100%)
ANA-IIF AC-19/AC-20 patterns	Anti-ribosomal *P*	50/59 (84.7%)
ENA screening positive/borderline	Antigen-specific ENA characterization	55/92 (59.8%)
Overall population	≥1 missed follow-up test	508/2207 (23.0%)

Abbreviations: ANA-IIF, antinuclear antibody indirect immunofluorescence; ENA, extractable nuclear antigen; AC, anti-cell pattern code; AMA-IIF, antimitochondrial antibody indirect immunofluorescence; Sp100, Sp100 nuclear antigen; gp210, nuclear pore glycoprotein 210; ribosomal P, ribosomal P protein.

**Table 3:** Serological yield of the simulated stepwise and upfront parallel strategies.

Strategy	Population	Screening-Level Signals *n* (%)	ENA-Specific Positive	CLIFT-Positive	Patients with Fully Characterized Finding *n* (%)
Stepwise	514	31 (6.0%)	13 (2.5%)	3 (0.6%)	15 (2.9%)
Upfront	514	47 (9.1%)	18 (3.5%)	5 (1.0%)	22 (4.3%)
*p*-value *		<0.001			0.016

* *p*-values and 95% confidence intervals (95% CI) were calculated using McNemar’s test for paired nominal data to compare the stepwise and upfront strategies (*N* = 514). The absolute risk difference for screening-level signals was +3.11% (95% CI: 1.62% to 4.61%), and for fully characterized findings, it was +1.36% (95% CI: 0.47% to 2.25%).

**Table 4:** Estimated laboratory workload of stepwise and upfront parallel strategies. The economic analysis was restricted to the core ENA/dsDNA component that differentiates the two simulated strategies. Pattern-driven follow-up assays triggered by ANA-IIF fluorescence patterns, including AMA-IIF, liver-specific antigen-specific assays, anti-ribosomal *P*, anti-DFS70, and anti-PCNA, were excluded.

Strategy	ANA-IIF	ENA Screening	Anti-dsDNA SPA	Antigen-Specific ENA Tests	CLIFT	Total Tests	Tests/Patient	Estimated Total Cost
Stepwise	514	222	222	152	13	1123	2.18	4440 €
Upfront	514	514	514	216	22	1780	3.46	7032 €
Incremental burden	+0	+292	+292	+64	+9	+657	+1.28	+2592 €

Abbreviations: ANA-IIF, antinuclear antibody indirect immunofluorescence; ENA, extractable nuclear antigen; dsDNA, double-stranded DNA; SPA, solid-phase assay; CLIFT, Crithidia luciliae immunofluorescence test.

## Data Availability

The individual-level data generated and analyzed during the current study are not publicly available due to privacy and ethical restrictions, as the combination of clinical referral information and laboratory variables could potentially allow patient re-identification. Aggregated data supporting the findings of this study are provided in the Supplementary Materials.

## Supplementary Materials

The following supporting information can be downloaded at: https://www.mdpi.com/article/10.3390/biomedicines14081815/s1, Table S1: Comparison of baseline laboratory and referral characteristics between the fully characterized subgroup and the remaining study cohort.
