# Supplementary material for: Real-World Evaluation of ANA Testing Pathways: Serological Incompleteness and Comparison of Stepwise Versus Upfront Parallel Reflex Strategies
Source: Biomedicines. 2026 Aug 12;14(8):1815. doi: 10.3390/biomedicines14081815 (PMC13509393; doi:10.3390/biomedicines14081815)
Supplement: Supplementary file 1 [file biomedicines-14-01815-s001.zip › biomedicines-4458194-supplementary.pdf]

Table S1. Comparison of baseline laboratory and referral characteristics between the fully characterized subgroup and the remaining study cohort.

ANA-IIF positive	913 (41.4%)	691 (40.8%)	222 (43.2%)
ANA-IIF negative	1294 (58.6%)	1002 (59.2%)	292 (56.8%)
**ANA-IIF titre distribution (ANA-IIF-positive patients)**			
1:160	655 (71.7%)	496 (71.8%)	159 (71.6%)
1:320	184 (20.2%)	139 (20.1%)	45 (20.3%)
≥1:640	74 (8.1%)	56 (8.1%)	18 (8.1%)
**Clinical indication categories**			
Musculoskeletal symptoms	567 (25.7%)	427 (25.2%)	140 (27.2%)
Non-classifiable indications	159 (7.2%)	121 (7.1%)	38 (7.4%)
Dermatological manifestations	167 (7.6%)	129 (7.6%)	38 (7.4%)
Hepatological/gastroenterological conditions	173 (7.8%)	136 (8.0%)	37 (7.2%)
Suspected systemic autoimmune disease	174 (7.9%)	131 (7.7%)	43 (8.4%)
Pulmonary/respiratory conditions	125 (5.7%)	94 (5.6%)	31 (6.0%)
Other indications	842 (38.2%)	655 (38.7%)	187 (36.4%)

Note: ANA-IIF titre percentages are calculated among ANA-IIF-positive patients (overall n = 913; remaining cohort n = 691; fully characterized subgroup n = 222). Clinical indication percentages use the corresponding cohort size as denominator. The overall cohort is shown for descriptive purposes only.
